# Detection of tumour heterogeneity in patients with advanced, metastatic castration-resistant prostate cancer on [^68^Ga]Ga-/[^18^F]F-PSMA-11/-1007, [^68^Ga]Ga-FAPI-46 and 2-[^18^F]FDG PET/CT: a pilot study

**DOI:** 10.1007/s00259-024-06891-8

**Published:** 2024-08-29

**Authors:** Kim M. Pabst, Riccardo Mei, Katharina Lückerath, Boris A. Hadaschik, Claudia Kesch, Josefine Rawitzer, Lukas Kessler, Luisa S. Bodensieck, Rainer Hamacher, Kelsey L. Pomykala, Stefano Fanti, Ken Herrmann, Wolfgang P. Fendler

**Affiliations:** 1 Department of Nuclear Medicine, West German Cancer Center, University Hospital Essen, Essen, Germany; 2 German Cancer Consortium (DKTK), Partner Site University Hospital Essen, Essen, Germany; 3 Division of Nuclear Medicine, IRCCS Azienda Ospedaliero-Universitaria Di Bologna, Bologna, Italy; 4 Department of Urology, West German Cancer Center, University Hospital Essen, Essen, Germany; 5 Institute of Pathology, University Hospital Essen, University of Duisburg-Essen, Essen, Germany; 6 Department of Radiology and Neuroradiology, University Hospital Essen, Essen, Germany; 7 Department of Medical Oncology, West German Cancer Center, University Hospital Essen, Essen, Germany; 8 Institute for AI in Medicine (IKIM), University Medicine Essen, Essen, Germany; 9 Cancer Research Center Cologne Essen (CCCE), University Medicine Essen, Essen, Germany

**Keywords:** Prostate cancer, MCRPC, ^68^Ga-FAPI, ^18^F-FDG, PSMA

## Abstract

**Purpose:**

In metastatic castration-resistant prostate cancer (mCRPC), some patients show low/absent PSMA expression in tumour lesions on positron emission tomography (PET) scans, indicating heterogeneity and heightened risk of non-response to PSMA-RLT (radioligand therapy). Imaging cancer-associated fibroblasts and glucose uptake may further characterise tumour heterogeneity in mCRPC patients. Here, we aimed to evaluate tumour heterogeneity and its potential implications for management in mCRPC patients assessed for PSMA-RLT using [^68^Ga]Ga-FAPI-46, 2-[^18^F]FDG and [^68^Ga]Ga-/[^18^F]F-PSMA-11/-1007 PET.

**Material and Methods:**

Patients with advanced, progressive mCRPC underwent clinical [^68^Ga]Ga-/[^18^F]F-PSMA-11/-1007, 2-[^18^F]FDG and [^68^Ga]Ga-FAPI-46 PET/CT to evaluate treatment with PSMA-directed RLT. Tumour detection/semiquantitative parameters were compared on a per-lesion/-region basis. Two phenotypes were defined: Criteria for the mixed phenotype were: (a) PSMA-negative findings for lymph node metastases ≥ 2.5 cm, any solid organ metastases ≥ 1.0 cm, or bone metastases with soft tissue component ≥ 1.0 cm, (b) low [^68^Ga]Ga-/[^18^F]F-PSMA-11/-1007 uptake and/or (c) balanced tumour uptake of all radioligands. The PSMA-dominant phenotype was assigned if the criteria were not met.

**Results:**

In ten patients, 472 lesions were detected on all imaging modalities (miTNM regions: M1b: 327 (69.3%), M1a: 95 (20.1%), N1: 26 (5.5%), M1c: 18 (3.8%), T: 5 (1.1%) and Tr: 1 (0.2%). [^68^Ga]Ga-/[^18^F]F-PSMA-11/-1007 (n = 453 (96.0%)) demonstrates the highest detection rate, followed by [^68^Ga]Ga-FAPI-46 (n = 268 (56.8%))/2-[^18^F]FDG (n = 241 (51.1%)). Semiquantitative uptake was highest for [^68^Ga]Ga-/[^18^F]F-PSMA-11/-1007 (mean SUV_max_ (interquartile range): 22.7 (22.5), vs. [^68^Ga]Ga-FAPI-46 (7.7 (3.7)) and 2-[^18^F]FDG (6.8 (4.7)). Seven/three patients were retrospectively assigned to the PSMA-dominant/mixed phenotype. Median overall survival was significantly longer for patients who underwent [^177^Lu]Lu-PSMA-617 RLT and were retrospectively assigned to the PSMA-dominant phenotype (19.7 vs. 9.3 months).

**Conclusion:**

Through whole-body imaging, we identify considerable inter- and intra-patient heterogeneity of mCRPC and potential imaging phenotypes. Regarding uptake and tumour detection, [^68^Ga]Ga-/[^18^F]F-PSMA-11/-1007 was superior to [^68^Ga]Ga-FAPI-46 and 2-[^18^F]FDG, while the latter two were comparable. Patients who underwent [^177^Lu]Lu-PSMA-617 RLT based on clinical-decision making had a longer overall survival and could be assigned to the PSMA-dominant phenotype.

**Graphical Abstract:**

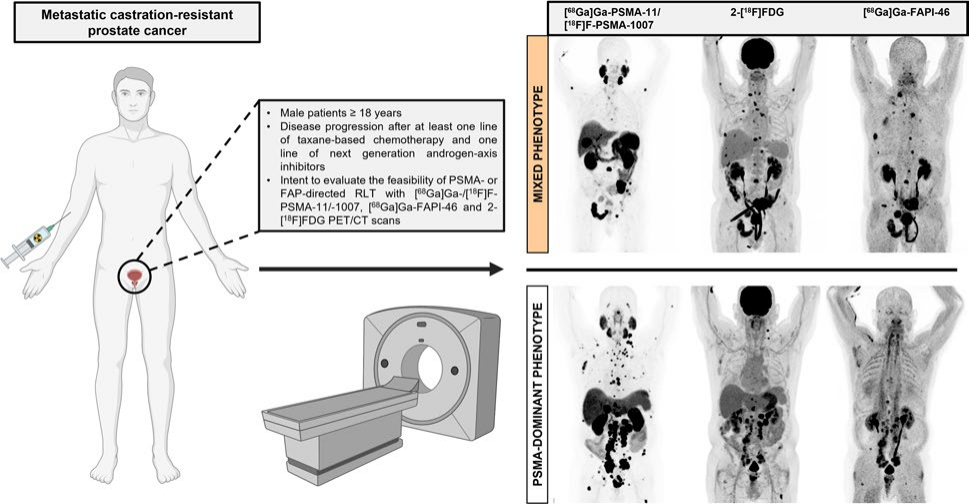

**Supplementary Information:**

The online version contains supplementary material available at 10.1007/s00259-024-06891-8.

## Introduction

Prostate cancer is the third most commonly diagnosed malignancy worldwide [1, 2]. Most metastatic prostate cancers are initially hormone-sensitive, but become resistant to therapy over time and develop into metastatic-castration resistant prostate cancer (mCRPC) [3]. [^177^Lu]Lu-PSMA-617 (prostate specific membrane antigen) radioligand therapy (RLT) is a favourable treatment option for patients with end-stage mCRPC [4]. To determine patient eligibility for RLT, [^68^Ga]Ga-/[^18^F]F-PSMA-11/-1007 positron emission tomography (PET)/computed tomography (CT) is performed to assess tumour PSMA-expression. However, the level of PSMA expression can vary significantly both intra- and inter-individually [5, 6]. Consequently, a subset of patients with low/absent PSMA expression is not amenable to [^177^Lu]Lu-PSMA-617 RLT. Moreover, due to intra-individual heterogeneous PSMA expression, treated patients may not respond sufficiently. Several factors, including prior/current therapies, epigenetic factors [5, 6], anatomic location of distant metastases [5], as well as variation in tumour dedifferentiation [7] and in the stromal component [8], may influence PSMA expression. To better stratify [^177^Lu]Lu-PSMA-617 RLT candidates and to support translation of new therapeutic options for patients with insufficient PSMA expression, a better understanding of tumour heterogeneity in mCRPC patients is needed.

In clinical routine, ^18^F-Fluorodeoxyglucose (2-[^18^F]FDG) PET may be used additionally to detect potential mismatch findings and thus help to identify less suitable candidates for [^177^Lu]Lu-PSMA-617 RLT [9, 10]. In addition to 2-[^18^F]FDG PET, somatostatin receptor expression has been investigated as a potential biomarker for neuroendocrine differentiation in mCRPC by using [^68^Ga]Ga-DOTATATE PET/CT. It was demonstrated that positive tumour lesions on [^68^Ga]Ga-DOTATATE PET are associated with a poorer prognosis [11]. However, the therapeutic aspect of this theranostic approach has only been investigated in a limited number of individual cases [12].

The novel radioligand [^68^Ga]Ga-FAPI-46 (fibroblast activation protein inhibitor) specifically targets the fibroblast activation protein (FAP), which is predominantly expressed by cancer-associated fibroblasts in the stroma of various solid tumours. FAP has been identified by genomic and immunohistochemical analyses as a target structure for imaging the tumour microenvironment in prostate cancer [13]. Furthermore, tissue analyses demonstrate that FAP expression increases with the advancement of disease, particularly in CRPC [14]. In addition, FAP appears to be a marker of neuroendocrine differentiation and is associated with a more aggressive course and shorter survival [15]. Initial applications of FAP-targeted imaging radioligands in prostate cancer patients, particularly those with low PSMA expression, have shown promising results [16–18]*.* Therefore, stromal imaging with [^68^Ga]Ga-FAPI-46 may be a useful tool to provide a comprehensive assessment of tumour heterogeneity, which has not yet been studied in detail.

The results of the limited number of FAP-directed RLT available to date are heterogeneous [19, 20]. However, Assadi et al. observed disease stabilisation in a prostate cancer patient after 4.5 months and a single cycle of [^177^Lu]Lu-DOTA-FAPi-46 (1.85 GBq) [20], raising the question of whether FAPI-RLT might be a potential option for PSMA-negative patients [21].

We hypothesise that combined [^68^Ga]Ga-/[^18^F]F-PSMA-11/-1007, 2-[^18^F]FDG and [^68^Ga]Ga-FAPI-46 PET imaging will reveal considerable imaging-based heterogeneity of mCRPC, with implications for patient management and outcome. In the present study, we compared [^68^Ga]Ga-/[^18^F]F-PSMA-11/-1007, 2-[^18^F]FDG and [^68^Ga]Ga-FAPI-46 tumour/stromal uptake in mCRPC patients undergoing screening for [^177^Lu]Lu-PSMA-617 RLT eligibility.

## Materials and methods

### Patient population

**Fig. 1:**
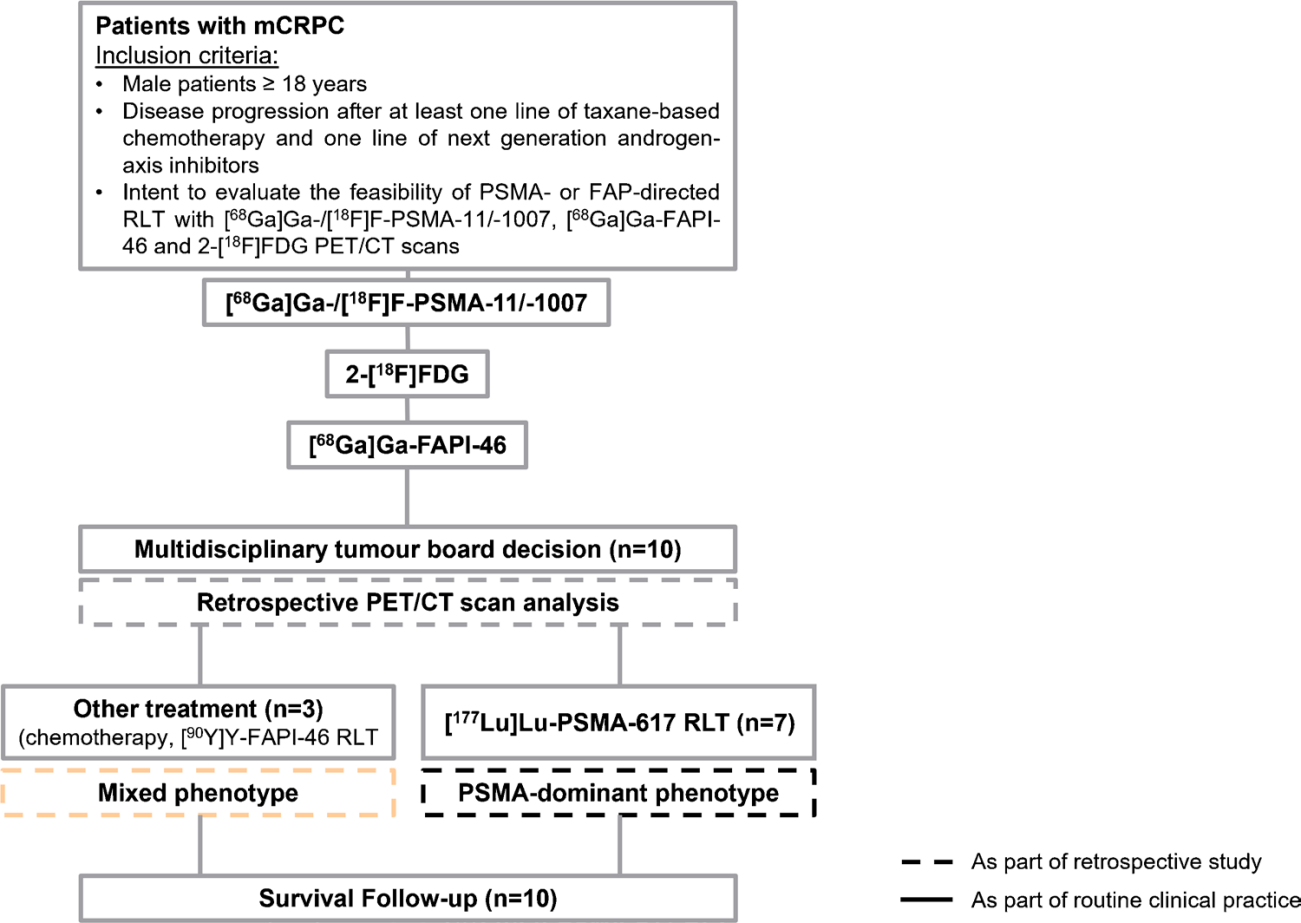
Flow of patients

The patient flowchart is shown in Fig. 1; an overview of PET scans is shown in Supplemental Fig. 1. This is a subgroup analysis of the ongoing observational trial (NCT04571086) at the University Hospital Essen. Between August 2021 and November 2021, 10 patients with mCRPC were included (2.5% of the entire trial). Before enrolment, patients gave written informed consent to undergo [^68^Ga]Ga-/[^18^F]F-PSMA-11/-1007, [^68^Ga]Ga-FAPI-46 and 2-[^18^F]FDG PET/CT for clinical assessment of PSMA- or FAP-directed RLT. Inclusion criteria were (a) male patients ≥ 18 years with advanced mCRPC, (b) disease progression after at least one line of chemotherapy and one line of next generation androgen-axis inhibitors, (c) screening for PSMA- and FAP-directed RLT. This study was approved by the local Ethics Committee (19–8991-BO and 20–9485-BO).

### Image acquisition

#### [^68^Ga]Ga-FAPI-46 PET/CT

The radiosynthesis of [^68^Ga]Ga-FAPI-46 was described previously [22]. Patients did not fast. The median activity administered intravenously was 81 MBq (interquartile range (IQR): 24.5 MBq). The median time from injection to acquisition was 15 min (IQR: 8.5 min). PET scans were performed on a PET/CT system (Vision, Siemens, Erlangen, Germany) in combination with low-dose CT without the application of intravenous contrast.

#### [^68^Ga]Ga-/[^18^F]F-PSMA-11/-1007 PET/CT

[^68^Ga]Ga-PSMA-11 was administered in *n* = 4 (40%) patients, [^18^F]F-PSMA-1007 in *n* = 6 (60%) patients. The median activity administered intravenously was 96.5 MBq (IQR: 25.8 MBq) for [^68^Ga]Ga-PSMA-11 and 307 MBq (IQR: 62.3 MBq) for [^18^F]F-PSMA-1007. The median time from injection to acquisition was 42 min (IQR: 10 min) for [^68^Ga]Ga-PSMA-11 and 83 min (IQR: 4 min) for [^18^F]F-PSMA-1007. Intravenous contrast was administered in 7 patients (70%) and PET scans were performed on a PET/CT system (mCT Biograph or Vision, Siemens, Erlangen, Germany). The PET protocol was in accordance with the international procedure standard for PSMA PET/CT for prostate cancer imaging [23].

#### 2-[^18^F]FDG PET/CT

The median injected activity of 2-[^18^F]FDG was 326 MBq (IQR: 75.7 MBq). The median time from injection to acquisition was 69.5 min (IQR: 17.3 min). Low-dose PET scans were performed on a PET/CT system (mCT Biograph or Vision, Siemens, Erlangen, Germany). The PET protocol complied with guidelines for tumour imaging [24].

### Image evaluation

For comparison of [^68^Ga]Ga-FAPI-46, 2-[^18^F]FDG and [^68^Ga]Ga-/[^18^F]F-PSMA-11/-1007 tumour uptake, a per-lesion-based analysis of the maximum and mean standardized uptake value (SUV_max_, SUV_mean_) and metabolic tumour volume was performed in consensus by two independent, blinded nuclear medicine physicians. For calculation of SUV_mean_ and metabolic tumour volume, volumes of interest were determined by an isocontour threshold of 41% of SUV_max_. Syngo.via software (Siemens Healthineers) was used for measurements of SUV and metabolic tumour volume.

### Detection efficacy

Detection efficacy was assessed through a per-lesion-based (with the exception of confluent lesions, each lesion was analysed individually)/-region-based (miTNM regions: T: primary tumour, Tr: local tumour recurrence, N1: Regional lymph nodes, M1a: Distant lymph nodes, M1b: Bone metastases, M1c: Visceral metastases) evaluation of [^68^Ga]Ga-FAPI-46, 2-[^18^F]FDG and [^68^Ga]Ga-/[^18^F]F-PSMA-11/-1007 PET [25]. Each detected lesion was considered positive, regardless of the imaging modality. On PET, focal uptake above the surrounding background level that was not attributable to physiological findings on CT was rated positive. Lesions visible on multiple imaging modalities were listed once. Follow-up imaging (CT, SPECT/CT or [^68^Ga]Ga-/[^18^F]F-PSMA-11/-1007 PET/CT) and clinical data including PSA levels were used as standard of truth.

### Imaging phenotype of patients

Two phenotypes (PSMA-dominant, mixed) were defined. For the mixed phenotype, the following criteria were used: (a) PSMA-negative findings for lymph node metastasis ≥ 2.5 cm in short axis or any solid organ metastasis ≥ 1.0 cm in short axis, or metastatic bone disease with a soft tissue component ≥ 1.0 cm in short axis [26], (b) low visual [^68^Ga]Ga-/[^18^F]F-PSMA-11/-1007 uptake (< healthy liver uptake for [^68^Ga]Ga-PSMA-11 [26] and < healthy splenic uptake for [^18^F]F-PSMA-1007 [27]) and/or (c) balanced tumour uptake of the three radioligands, defined as a deviation of the mean SUV_mean_ of all lesions detected by 2-[^18^F]FDG and [^68^Ga]Ga-FAPI-46 PET of ≤ 30% of the mean SUV_mean_ of [^68^Ga]Ga-/[^18^F]F-PSMA-11/-1007 PET. The PSMA-dominant phenotype was assigned if the criteria were not met.

### Statistical analysis

Descriptive statistics and individual patient data are reported. Median, mean, IQR, and range were used for continuous data. Normal distribution was tested by Shapiro–Wilk test and could not be confirmed (*p* < 0.001). Therefore, in order to compare semiquantitative parameters, Friedman's test with post hoc test was applied. The Mann–Whitney U test was performed to compare semiquantitative parameters between the two phenotypes. Overall survival was analysed by log-rank test. To demonstrate the results, bar charts, Kaplan–Meier curves and heat maps were used for visualization. A *p*-value < 0.05 was considered statistically significant. All analyses were performed using SPSS Statistics (version 27.0; IBM) and Prism (version 9.1.0; GraphPad Software).

## Results

### Patient characteristics

Overall, 10 patients with advanced mCRPC were enrolled. The median age was 71 years (range: 62–86 years). The median PSA level at the time of [^68^Ga]Ga-/[^18^F]F-PSMA-11/-1007 PET/CT was 156 ng/mL (range: 2.5–747 ng/mL). All patients had received prior chemotherapy and at least one line of next generation androgen-axis inhibitors. Prior to screening for eligibility for [^177^Lu]Lu-PSMA-617 RLT, *n* = 7 (70%) patients had bone and *n* = 2 patients had visceral metastases (20%). Patients’ characteristics are summarized in Table 1.

**Table 1:** Patients’ characteristics

Imaging phenotype	Patient No	Age	Initial Diagnosis	Gleason-Score	miTNM (PET/CT)	PSA (ng/mL)	Max. Time (days) between PET/CT scans	Prior systemic therapies	Therapy after PET/CT scans
PSMA-dominant	2	76	2012	5 + 4 = 9	T0 N2 M1a	170	9	ADT, Abiraterone, Enzalutamide, Docetaxel	[^177^Lu]Lu-PSMA-617 RLT
	3	70	2016	n.a	T2m N2 M1a	747	1	ADT, Abiraterone, Enzalutamide, Docetaxel	[^177^Lu]Lu-PSMA-617 planned*
	4	63	2019	4 + 5 = 9	T3b N2 M1b	712	1	ADT, Abiraterone, Enzalutamide, Docetaxel, Cabazitaxel	[^177^Lu]Lu-PSMA-617 RLT
	6	86	07/2010	5 + 3 = 8	T0 N1 M1a M1c	162	48	ADT, Abiraterone, Enzalutamide, Bicalutamide, Docetaxel	[^177^Lu]Lu-PSMA-617 RLT
	8	62	2015	4 + 5 = 9	Tr N2 M1a M1b	27	2	ADT, Abiraterone, Enzalutamide, Docetaxel	[^177^Lu]Lu-PSMA-617 RLT
	9	78	2012	3 + 5 = 8	T0 N2 M1 M1b	499	34	ADT, Abiraterone, Docetaxel	[^177^Lu]Lu-PSMA-617 RLT
	10	62	2018	4 + 4 = 8	T2m N0 M1b	7	1	ADT, Abiraterone, Docetaxel, Cabazitaxel	[^177^Lu]Lu-PSMA-617 RLT
Mixed	1	66	2019	4 + 4 = 8	T2 N1 M1b M1c	3	13	ADT, Abiraterone, Docetaxel	Chemotherapy
	5	81	2018	4 + 4 = 8	T3b N0 M1b	60	1	ADT, Enzalutamide, Docetaxel, ^223^Radium	[^90^Y]Y-FAPI-46 RLT
	7	71	2017	4 + 3 = 7b	T0 N2 M1a M1b	149	8	ADT, Abiraterone, Enzalutamide, Docetaxel	Chemotherapy

Individual patient characteristics, categorized by imaging phenotype (PSMA-dominant, mixed). Molecular imaging TNM system, a standardized reporting framework for PSMA-ligand PET/CT, was applied. *[^177^Lu] Lu-PSMA-617 RLT was not conducted due to disease progression leading to the patient's demise. *ADT* androgen-deprivation therapy

### Detection efficacy

**Fig. 2:**
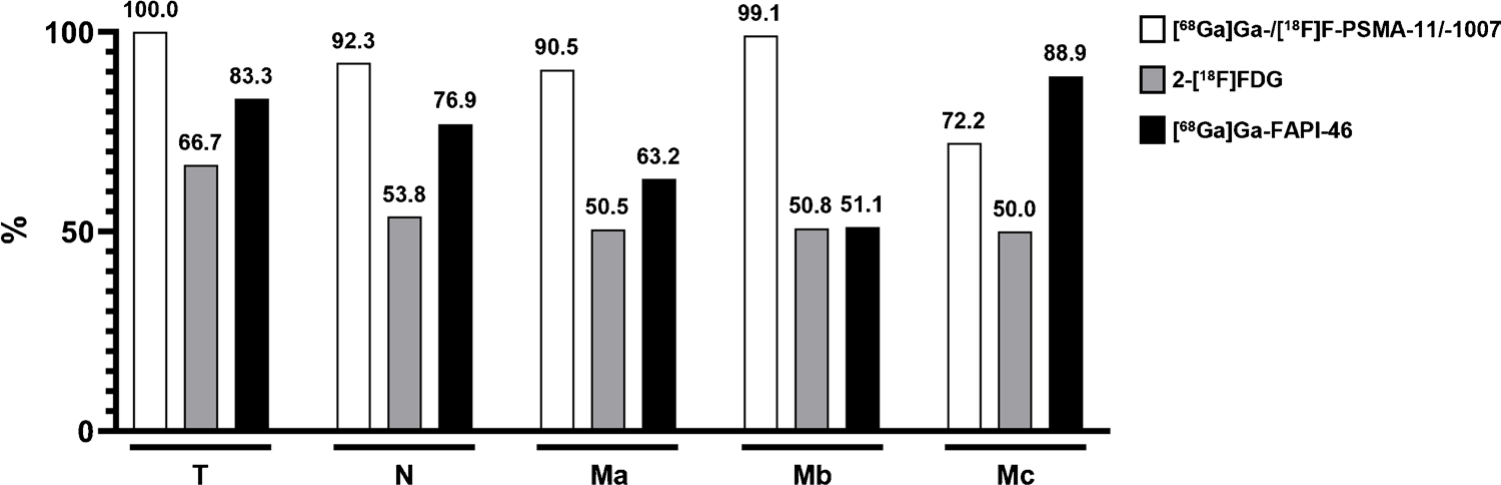
**Per-region-based detection efficacy.** Per-region-based analysis of detection efficacy (T: primary tumour/local tumour recurrence, N1: regional lymph node metastases, M1a: distant lymph node metastases, M1b: bone metastases, M1c: visceral metastases) for [^68^Ga]Ga-/[^18^F]F-PSMA-11/-1007 (white), 2-[^18^F]FDG (grey) and [^68^Ga]Ga-FAPI-46 PET (black bars)

**Fig. 3:**
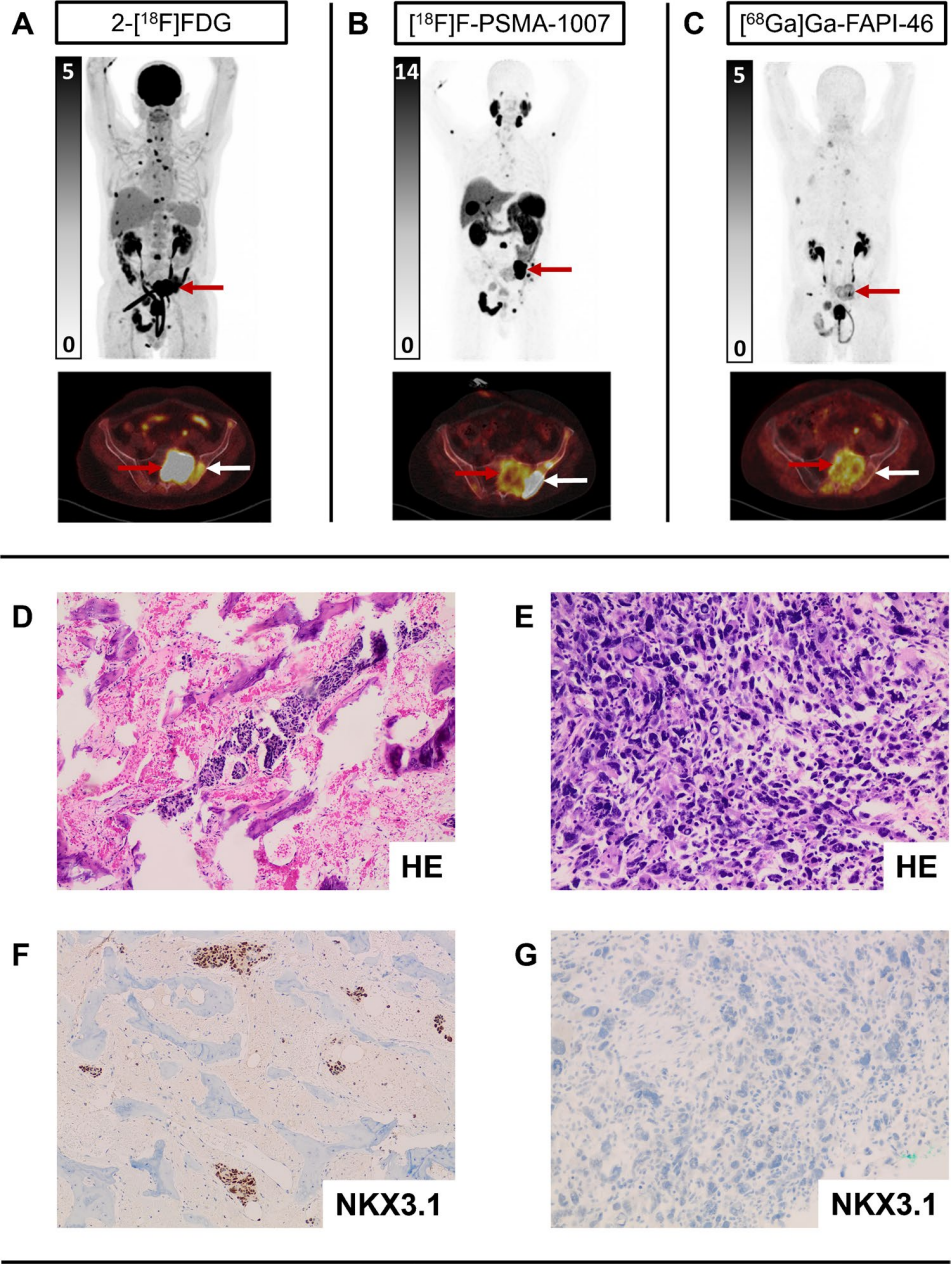
**Heterogeneity of radioligand uptake in patient no. 1.** Example of heterogeneity regarding uptake pattern in a pelvic bone metastasis in patient no 1. 2-[^18^F]FDG uptake was highest in the os sacrum (**A**, red arrow) and low to moderate uptake in the os ilium (**A**, white arrow). [^18^F]F-PSMA-1007 uptake was highest in the os ilium (**B**, white arrow) and lower in the os sacrum (**B**, red arrow). [^68^Ga]Ga-FAPI-46 uptake was only moderate in the os sacrum (**C**, red arrow) and nearly absent in the os ilium (**C**, white arrow). After the PET-scans, a biopsy of both regions was performed, revealing a metastasis of prostate adenocarcinoma in the os ilium (**D**, **F**; 100-fold magnification) and a metastasis of dedifferentiated prostate cancer in the os sacrum (**E**,**G**; 400-fold magnification) which was confirmed by immunohistochemical staining for NKX3.1 (detection of NKX3.1 (brown) in **F**, absence of NKX3.1 in **G**). The patient received chemotherapy and died 4 months after imaging

A total of 472 lesions were detected across all imaging modalities, ranging from a minimum of 16 to 115 lesions per patient. In descending order of frequency, the lesions were located in the following regions: bones: 327 (69.3%), distant lymph nodes: 95 (20.1%), regional lymph nodes: 26 (5.5%), visceral metastases: 18 (3.8%), primary tumour: 5 (1.1%), and local tumour recurrence: 1 (0.2%). Overall, [^68^Ga]Ga-/[^18^F]F-PSMA-11/-1007 (*n* = 453 (96.0%)) had the highest detection rate, followed by [^68^Ga]Ga-FAPI-46 (*n* = 268 (56.8%)) and 2-[^18^F]FDG (*n* = 241 (51.1%)). In a per-region-based analysis, [^68^Ga]Ga-/[^18^F]F-PSMA-11/-1007 was superior to the other two tracers, except for visceral lesions where [^68^Ga]Ga-FAPI-46 had the highest detection rate (further details can be found in Fig. 2). In a per-lesion-based analysis, 6 lesions were only 2-[^18^F]FDG positive (N1: 2, M1a: 1, M1b: 1, M1c: 2) and 5 lesions were only [^68^Ga]Ga-FAPI-46 positive (M1a: 3, M1b: 1, M1c: 1). Mismatch findings are summarised in Table 2. An example of a heterogeneous uptake pattern is shown in Fig. 3.

**Table 2:** Mismatch findings

	[^68^Ga]Ga-/[^18^F]F-PSMA-11/-1007 positive *n* (%)	[^68^Ga]Ga-/[^18^F]F-PSMA-11/-1007 negative *n* (%)	Overall *n* (%)
[^68^Ga]Ga-FAPI-46 positive/ 2-[^18^F]FDG positive *n* (%)	207 (44%)	5 (1%)	212 (45%)
[^68^Ga]Ga-FAPI-46 positive / 2-[^18^F]FDG negative *n* (%)	43 (9%)	13 (3%)	56 (12%)
[^68^Ga]Ga-FAPI-46 negative / 2-[^18^F]FDG positive *n* (%)	25 (5%)	4 (1%)	29 (6%)
[^68^Ga]Ga-FAPI-46 negative / 2-[^18^F]FDG negative *n* (%)	175 (37%)	0 (0%)	175 (37%)
Overall *n* (%)	450 (95%)	22 (5%)	472 (100%)

Per-lesion-based mismatch findings (*n* (%)) on [^68^Ga]Ga-/[^18^F]F-PSMA-11/-1007, 2-[^18^F]FDG and [^68^Ga]Ga-FAPI-46 PET

### Semiquantitative analysis of tumour uptake

**Fig. 4:**
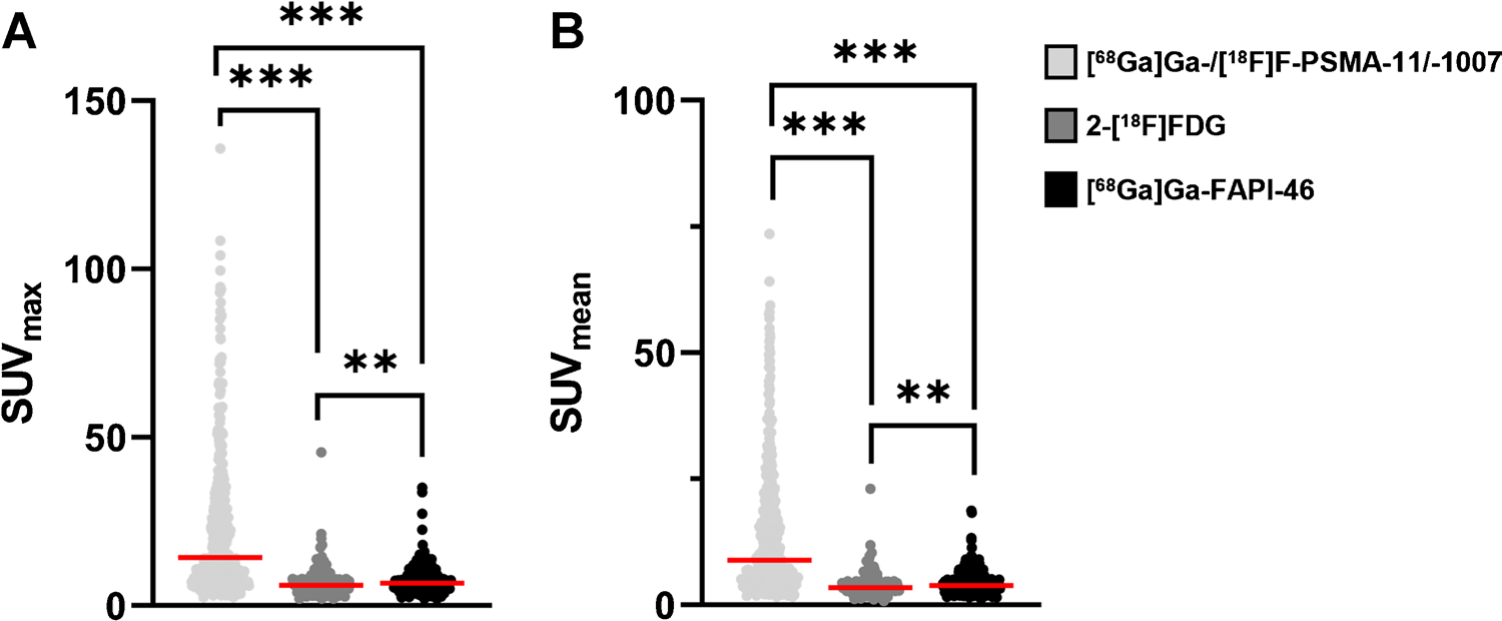
**Per-lesion-based comparison of SUV**_**max**_ **and SUV**_**mean**_ **between [**^**68**^**Ga]Ga-/[**^**18**^**F]F-PSMA-11/-1007, 2-[**^**18**^**F]FDG and [**^**68**^**Ga]Ga-FAPI-46.** In the absence of a normal distribution, the Friedman test was used for pairwise comparisons. *N* = 207 lesions were compared demonstrating uptake of all three radioligands. ***: *p* < 0.001, **: *p* < 0.01

For semiquantitative analysis, the SUV_max_ and SUV_mean_ values of the three radioligands in tumour lesions were compared. In a per-lesion-based analysis, both parameters were significantly higher for [^68^Ga]Ga-/[^18^F]F-PSMA-11/-1007 (mean ± standard deviation (SD): SUV_max_ 22.7 ± 21.3, SUV_mean_ 14.0 ± 12.8) vs. 2-[^18^F]FDG (SUV_max_ 6.8 ± 4.1, SUV_mean_ 4.0 ± 2.2) and [^68^Ga]Ga-FAPI-46 PET (SUV_max_ 7.7 ± 4.4, SUV_mean_ 4.5 ± 2.5) (all *p*-values < 0.001). [^68^Ga]Ga-FAPI-46 uptake (SUV_max_, SUV_mean_) was significantly higher compared to 2-[^18^F]FDG (*p* = 0.003/*p* = 0.001). Further details are shown in Fig. 4.

Similarly, in a per-patient-based analysis, uptake was significantly higher for [^68^Ga]Ga-/[^18^F]F-PSMA-11/-1007 (mean ± SD: SUV_max_ 19.1 ± 9.7, SUV_mean_ 11.7 ± 6.1) vs. 2-[^18^F]FDG (SUV_max_ 6.7 ± 1.2, *p* = 0.001, SUV_mean_ 3.9 ± 0.7, *p* = 0.002) and [^68^Ga]Ga-FAPI-46 PET (SUV_max_ 7.7 ± 1.7, *p* = 0.005, SUV_mean_ 4.5 ± 1.0, / *p* = 0,022). [^68^Ga]Ga-FAPI-46 and 2-[^18^F]FDG uptake did not differ significantly (SUV_max_ *p* = 1.0, SUV_mean_ *p* = 1.0).

### Patient imaging phenotypes

**Fig. 5:**
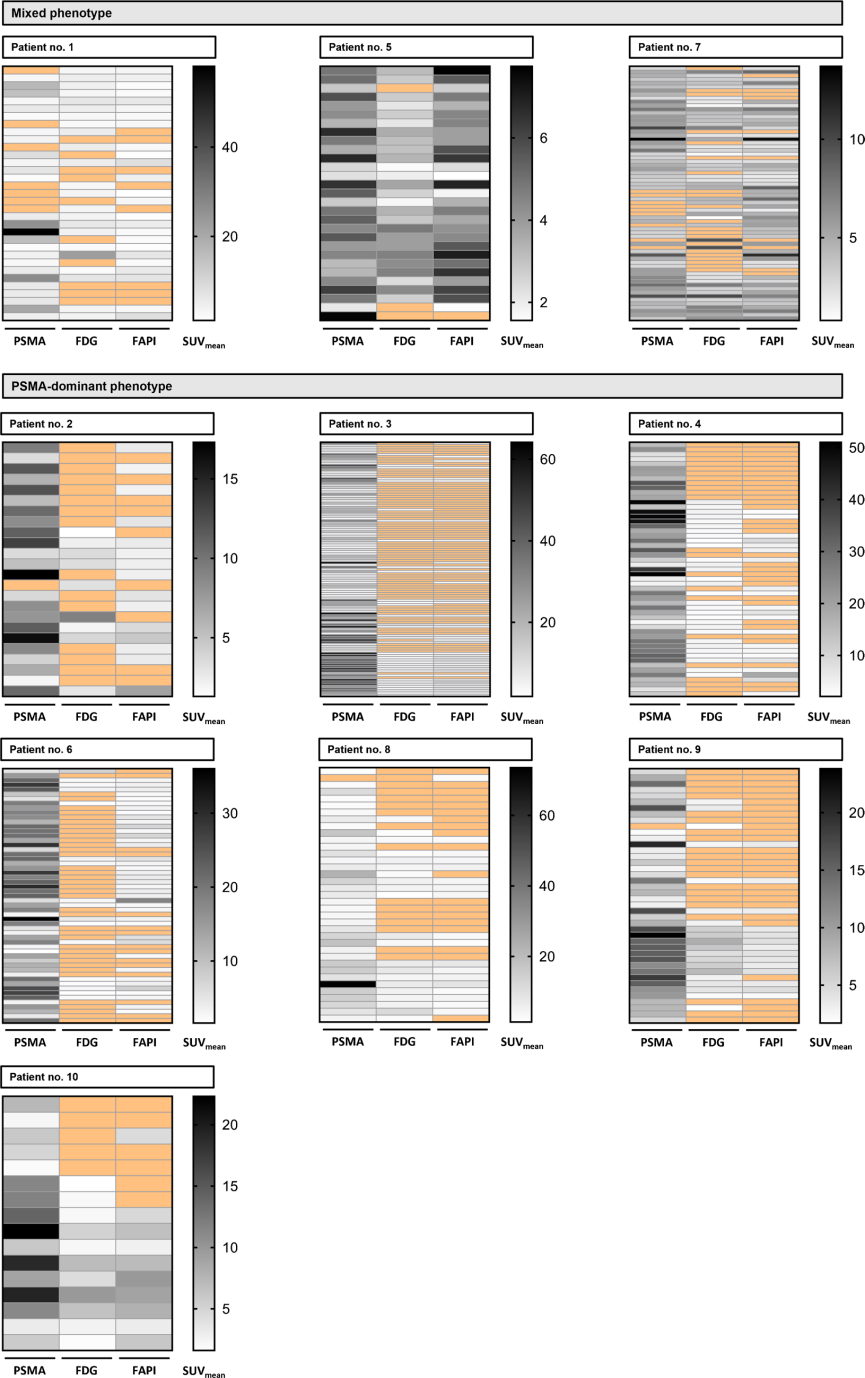
**Per-lesion-based heat maps (SUV**_**mean**_**) to demonstrate heterogeneous uptake and imaging-phenotypes of mCRPC.** SUV_mean-_based heat maps compared uptake of the three radioligands [^68^Ga]Ga-/[^18^F]F-PSMA-11/-1007, 2-[^18^F]FDG and [^68^Ga]Ga-FAPI-46 in each detected tumour lesion (each row corresponds to one lesion) and defined the imaging phenotype (PSMA-dominant vs. mixed). Negative lesions according to VISION criteria for [^68^Ga]Ga-/[^18^F]F-PSMA-11/-1007 and negative lesions (uptake equal to surrounding background) for 2-[^18^F]FDG and [^68^Ga]Ga-FAPI-46 [19] are shown in orange boxes, while lesions with tracer uptake are shown in grey scale reflecting the SUV_mean_ of the lesion. Note that the scale for the SUV_mean_ differs between patients to allow visualisation of intra-individual variation in the uptake of the three different radiotracers

Three patients had a mixed imaging phenotype and seven patients had a PSMA-dominant phenotype. Within the mixed phenotype cohort, patients 1 and 7 had relevant mismatch findings, which are exclusion criteria for [^177^Lu]Lu-PSMA-617 RLT according to the VISION trial (NCT03511664, [19]). In addition, patient 7 showed a balanced tumour uptake with a mean deviation from the SUV_mean_ of [^68^Ga]Ga-PSMA-11 PET in the other two PET modalities ([^68^Ga]Ga-FAPI-46: -9.8%, 2-[^18^F]FDG: -19.6%). Although patient 5 showed no mismatch findings, PSMA expression in the tumour lesions was low, predominantly < healthy liver, and tumour uptake of the three radioligands was balanced (mean deviation from the SUV_mean_ of [^68^Ga]Ga-PSMA-11 PET: [^68^Ga]Ga-FAPI-46: + 2.2%, 2-[^18^F]FDG: -28.9%), making him an unsuitable candidate for [^177^Lu]Lu-PSMA-617 RLT. Individual per-lesion-based heat maps of SUV_mean_ demonstrate considerable heterogeneity of radiotracer uptake (Fig. 5).

Average [^68^Ga]Ga-/[^18^F]F-PSMA-11/-1007 uptake was SUV_max_ 20.7 ± 10.1 and SUV_mean_ 13.9 ± 5.9 for the PSMA-dominant phenotype versus SUV_max_ 11.1 ± 4.8 and SUV_mean_ 6.6 ± 3.0 for the mixed phenotype. For 2-[^18^F]FDG, average SUV_max_ and SUV_mean_ were 6.5 ± 1.1 and 3.9 ± 0.6 for the PSMA-dominant and 7.1 ± 1.6 and 4.1 ± 0.9 for the mixed phenotype. [^68^Ga]Ga-FAPI-46 imaging resulted in an average SUV_max_ of 7.8 ± 2.0 and SUV_mean_ of 4.5 ± 1.2 in the PSMA-dominant vs. 7.2 ± 0.7 and 4.5 ± 0.7 in the mixed phenotype.

PSA levels in the PSMA-dominant subgroup averaged 332 ng/mL ± 315.8 ng/mL and in the mixed subgroup 70.5 ng/mL ± 73.8 ng/mL.

### Treatment and follow-up

All patients treated with [^177^Lu]Lu-PSMA-617 RLT in this cohort demonstrated retrospectively a PSMA-dominant phenotype (*n* = 7 (one patient was not treated due to disease progression leading to the patient's demise). Patients deemed ineligible for [^177^Lu]Lu-PSMA-617 RLT after clinical evaluation received chemotherapy (*n* = 2) or [^90^Y]Y-FAPI-46 RLT (*n* = 1) and could be retrospectively assigned to the mixed phenotype.

**Fig. 6:**
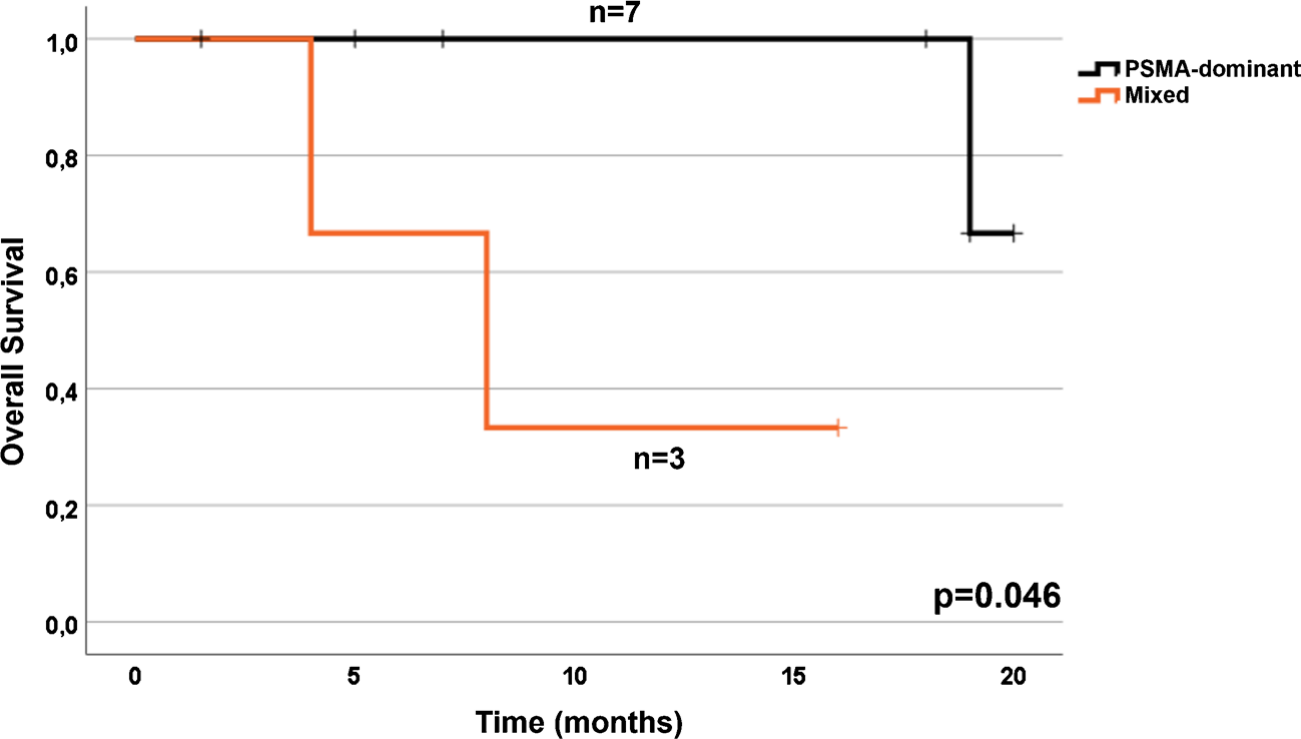
**Kaplan–Meier curves of overall survival by imaging phenotype (PSMA-dominant vs. mixed).** Patients with mixed phenotype (orange) demonstrates a significantly lower median overall survival of 9.3 months compared to patients with PSMA-dominant phenotype (black; 19.7 months)

The median follow-up was 12 months (± 7.3 months (standard deviation)). Patient with a PSMA-dominant phenotype had significantly longer overall survival when compared to patients with mixed phenotype (19.7 months vs. 9.3 months, *p* = 0.046, Fig. 6).

## Discussion

Prostate cancer is a heterogeneous disease in which the role of molecular imaging plays a central role, especially in mCRPC. With the approval and increasing use of [^177^Lu]Lu-PSMA-617 RLT, it has become apparent that low or heterogeneous PSMA expression may compromise the efficacy of RLT and be associated with short overall survival [26, 27], for example due to insufficient target dose to tumour lesions with low PSMA expression. There is currently an unmet clinical need to better understand tumour heterogeneity and how it can potentially be overcome or at least be managed.

The presence of PSMA-negative/FDG-positive tumour lesions has been demonstrated to serve as a negative predictor of overall survival in patients with mCRPC [28, 29]. Consequently, 2-[^18^F]FDG PET/CT has been included in the exclusion criteria for certain prospective studies [10]. Nevertheless, there is no theranostic counterpart for 2-[^18^F]FDG that enables therapeutic application for PSMA-negative mCRPC.

In recent years, the tumour microenvironment has become increasingly important in tumour diagnosis and therapy. FAP, which occurs predominantly in the tumour stroma, appears to be a potential marker for a more aggressive course and shorter survival in prostate cancer, similar to 2-[^18^F]FDG [15]. To date, a limited number of cases have been reported using [^68^Ga]Ga-FAPI-46 PET/CT in mCRPC patients, showing promising results particularly in patients with low PSMA expression, suggesting a potential clinical relevance [14, 16–18]. Therefore, we aimed to image tumour heterogeneity in a limited cohort of mCRPC patients as part of the eligibility assessment for [^177^Lu]Lu-PSMA-617 RLT using [^68^Ga]Ga-/[^18^F]F-PSMA-11/1007 to assess PSMA expression, 2-[^18^F]FDG for glucose uptake and [^68^Ga]Ga-FAPI-46 for FAP expression, and to identify potential descriptive imaging phenotypes to support therapeutic decision making.

Our findings demonstrated both intra- and inter-patient heterogeneity within one radioligand and across different radioligand modalities. On average, the SUV values on [^68^Ga]Ga-FAPI-46 and 2-[^18^F]FDG PET are comparable, while PSMA expression was significantly higher, as expected. However, detection rates for [^68^Ga]Ga-FAPI-46 were higher compared to 2-[^18^F]FDG, while both radioligands were inferior to [^68^Ga]Ga-/[^18^F]F-PSMA-11/-1007, raising the question of whether FAP-directed imaging could replace 2-[^18^F]FDG PET/CT in the evaluation of [^177^Lu]Lu-PSMA-617 RLT. Due to the small patient cohort, the results should initially be considered exploratory and verified in larger cohorts.

We identified two potential descriptive imaging phenotypes (PSMA-dominant and mixed) based on uptake behaviour in tumour lesions and mismatch findings according to current clinical criteria for [^177^Lu]Lu-PSMA-617 RLT [26, 27]. All patients who received [^177^Lu]Lu-PSMA-617 RLT could retrospectively be assigned to the PSMA-dominant phenotype. Patients who were deemed ineligible for [^177^Lu]Lu-PSMA-617 RLT met the criteria for the mixed phenotype, respectively. In particular, there was a significant difference in PSMA expression between the descriptive imaging phenotypes, while glucose uptake and FAP expression were largely similar.

Although all patients who underwent [^177^Lu]Lu-PSMA-617 RLT could be assigned to the PSMA-dominant phenotype, there were inter- and intra-individual differences in PSMA expression, which may be associated with response to RLT (Fig. 5). As an example, patient no. 8 presented with low initial SUV_mean_ values on [^68^Ga]Ga-PSMA-11 PET images and progressed after 2 cycles of [^177^Lu]Lu-PSMA-617 RLT. This resulted in discontinuation of RLT and switch to chemotherapy with cabazitaxel. This is in line with post-hoc findings of the VISION trial demonstrating lower response rates in patients with low initial SUV_mean_ [30]. Although we identify inter- and intra-individual heterogeneity, larger patient populations are needed to better understand these differences.

Patients with the mixed phenotype did not receive [^177^Lu]Lu-PSMA-617 RLT clinically. In the absence of alternative therapies and with sufficient FAP expression, FAP-directed RLT has been mentioned as a potential treatment in several case reports [16, 17] and Assadi et al. reported a stabilisation of tumour disease in one mCRPC patient using [^177^Lu]Lu-FAPI-46 RLT [20]. Patient no. 5, who presented with a mixed phenotype and high FAP expression in metastases, was considered clinically ineligible for [^177^Lu]Lu-PSMA-617 RLT and was treated with [^90^Y]Y-FAPI-46 RLT instead. The decision of the multidisciplinary tumour board to treat with [^90^Y]Y-FAPI-46 RLT was made because all approved treatment options had been exhausted. The patient received two cycles with 3.7 GBq and 7.4 GBq [^90^Y]Y-FAPI-46 six weeks apart. Restaging revealed progressive disease according to RECIST 1.1. Nevertheless, [^90^Y]Y-FAPI-46 RLT was feasible and well tolerated, which was described previously [19]. Although treatment with [^90^Y]Y-FAPI-46 did not result in tumour stabilisation/response, one case of stabilisation has been described with [^177^Lu]Lu-FAPI-46 RLT [20]. Optimized FAP-targeting RLT may become a therapeutic option to address PSMA-negative lesions/lesions with low PSMA expression and high FAP expression. However, the application of [^90^Y]Y-FAPI-46 is expected to play only a subordinate role in a limited subset of mCRPC patients due to the continued superiority of PSMA expression.

All patients who were clinically eligible for [^177^Lu]Lu-PSMA-617 RLT demonstrated collectively a longer overall survival compared to the subgroup of patients who were ineligible for [^177^Lu]Lu-PSMA-617 RLT, in accordance with previous publications [26]. The short median overall survival of 9.3 months in the mixed phenotype group emphasizes the prognostic relevance of PSMA-negative mismatch findings and low PSMA expression (26, 31). This may be explained by a lower tumour absorbed dose during [^177^Lu]Lu-PSMA-617 RLT in lesions with low PSMA expression, as well as potential differences in tumour biology reflected by the descriptive imaging phenotypes, which are currently not fully understood. A case example (Fig. 4) with an available biopsy performed shortly after the performed PET/CT scans provides a potential explanation for tumour heterogeneity: Tumour dedifferentiation was associated with loss of PSMA and PSMA-/FDG + /FAP + mismatch on PET scans. Further studies are needed to better comprehend these aspects.

A notable discrepancy was observed in the PSA values between the two phenotypes (PSMA-dominant: 332 ng/mL vs. mixed: 70.5 ng/mL). Due to the limited patient population, the statistical power is constrained. However, it may be associated with a lower tumour burden (Supplemental Fig. 1) on one hand and a dedifferentiation of the tumour and a decoupling of the PSA value in the mixed phenotype group on the other.

This study comes with limitations. This is a small study potentially lacking power to detect the true extent of heterogeneity. Flow of patients, including the time interval between PET/CT scans, follow-up imaging and PSA measurements, varied due to the retrospective nature of the study. Retrospective assessment may have introduced selection bias and mis-classification or information bias. Therefore, these results should be considered exploratory, and definitive conclusions should be based on future prospective evidence and mechanistic work-up.

In summary, the clinically applied criteria can be used to categorise two phenotypes that differ in terms of further treatment strategy and survival. However, it became evident that the clinical PET/CT criteria do not take all factors into account, as individual patients do not benefit from PSMA-RLT despite fulfilling all criteria.Nevertheless, the current findings indicate that [^68^Ga]Ga-FAPI-46 may be a viable alternative to 2-[^18^F]FDG, particularly in the context of a potential theranostic approach.

## Conclusion

Through PSMA-, glucose uptake- and FAP-directed whole body imaging, we find considerable inter- and intra-patient heterogeneity in mCRPC. [^68^Ga]Ga-/[^18^F]F-PSMA-11/-1007 uptake and tumour detection efficacy were superior to those of [^68^Ga]Ga-FAPI-46 and 2-[^18^F]FDG PET in all patients, while the latter two were comparable. However, we identify two potential imaging phenotypes, PSMA-dominant versus mixed disease, in which [^68^Ga]Ga-/[^18^F]F-PSMA-11/-1007 uptake is low and/or uptake of the three tracers is balanced. Patients who underwent [^177^Lu]Lu-PSMA-617 RLT based on clinical-decision making had a longer overall survival and could be assigned to the PSMA-dominant phenotype, compared to patients ineligible for [^177^Lu]Lu-PSMA-617 RLT who received chemotherapy or [^90^Y]Y-FAPI-46 RLT (mixed phenotype).

## Supplementary Information

Below is the link to the electronic supplementary material.

Supplementary file1 (DOCX 2703 KB)

## Data Availability

The datasets generated during and/or analysed during the current study are available from the corresponding author on reasonable request.

## Abbreviations

ADT: Androgen-deprivation therapy
FAP: Fibroblast activation protein
FAPI: Fibroblast activation protein inhibitor
2-[^18^F]FDG: ^18^F-Fluorodeoxyglucose
IQR: Interquartile range
M1a: Distant lymph node metastases
M1b: Bone metastases
M1c: Visceral metastases
mCRPC: Metastatic, castration-resistant prostate cancer
N1: Lymph node metastases
PSMA: Prostate specific membrane antigen
RLT: Radioligand therapy
SD: Standard deviation
SUV_max_: Maximum Standardized uptake value
SUV_mean_: Mean Standardized uptake value
T: Primary tumour
Tr: Local tumour recurrence
