# Supplementary material for: Detection of tumour heterogeneity in patients with advanced, metastatic castration-resistant prostate cancer on [68Ga]Ga-/[18F]F-PSMA-11/-1007, [68Ga]Ga-FAPI-46 and 2-[18F]FDG PET/CT: a pilot study
Source: Eur J Nucl Med Mol Imaging. 2024 Aug 29;52(1):342–53. doi: 10.1007/s00259-024-06891-8 (PMC11599349; doi:10.1007/s00259-024-06891-8)
Supplement: Supplementary file 1 — Supplementary file1 (DOCX 2703 KB) [file 259_2024_6891_MOESM1_ESM.docx]

**SUPPLEMENTAL FIGURES**


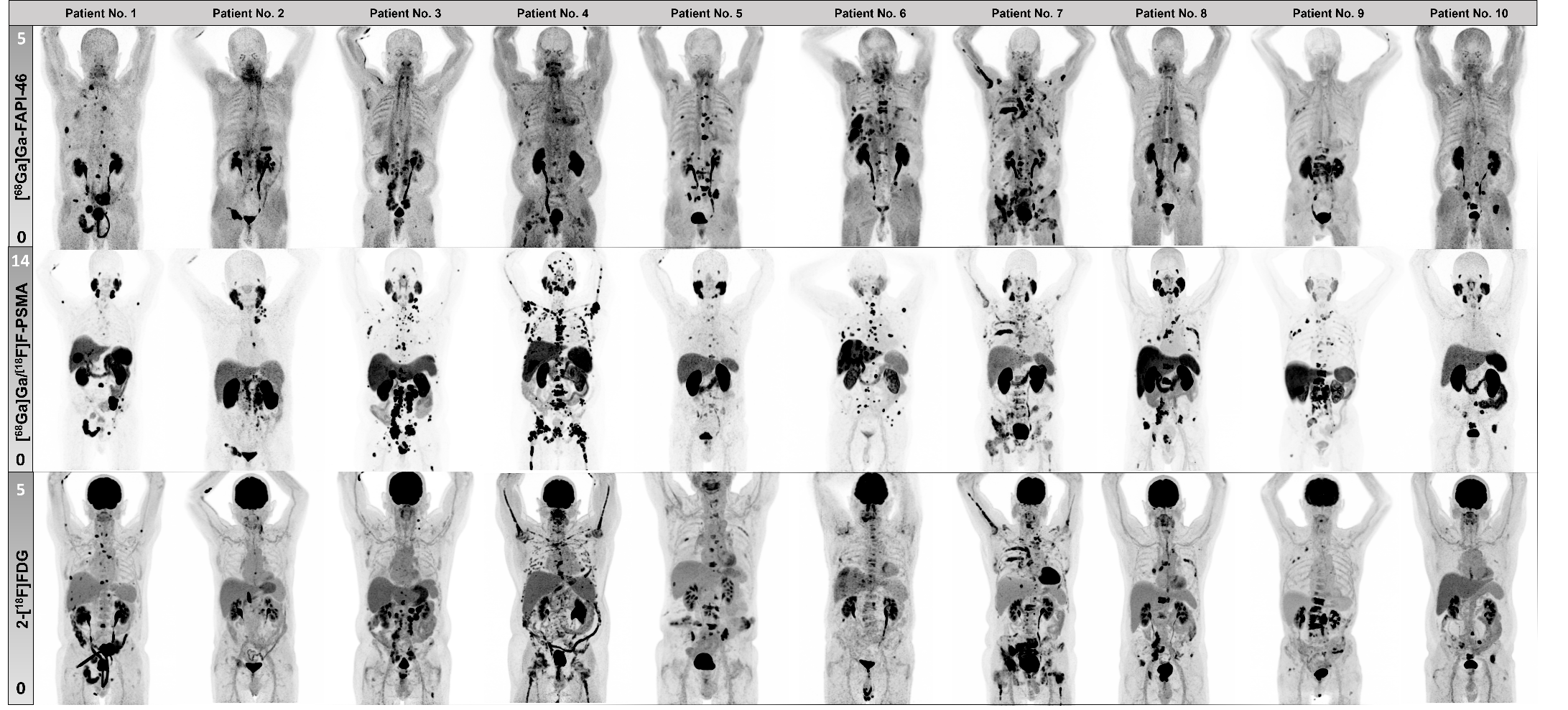
 **Supplemental Figure 1: Overview of Imaging Results in all Patients.** Maximum intensity projections of all patients, including [^68^Ga]Ga-/[^18^F]F-PSMA-11/-1007, 2-[^18^F]FDG and [^68^Ga]Ga-FAPI-46 PET images.

**GRAPHICAL ABSTRACT**

**
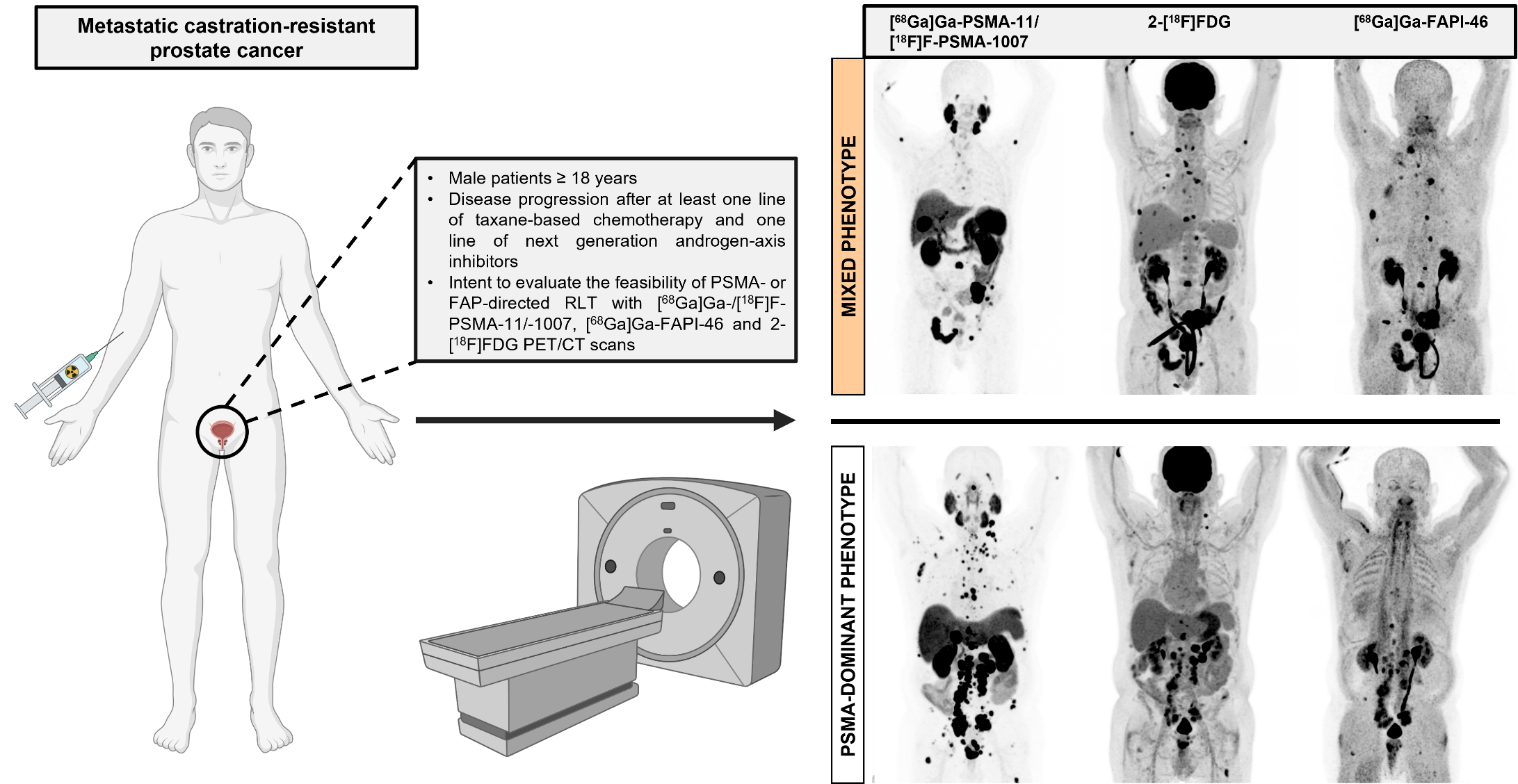
**
